# The Influence of Fall Direction and Hip Protector on Fracture Risk: FE Model Predictions Driven by Experimental Data

**DOI:** 10.1007/s10439-022-02917-0

**Published:** 2022-02-07

**Authors:** Ellie S. Galliker, Andrew C. Laing, Stephen J. Ferguson, Benedikt Helgason, Ingmar Fleps

**Affiliations:** 1 Institute for Biomechanics, ETH-Zurich, Zurich, Switzerland; 2 Department of Kinesiology and Health Sciences, University of Waterloo, Waterloo, Canada

**Keywords:** Femur, Bone, Finite element model, Impact, Hip protector, Elderly, Impact direction

## Abstract

Hip fractures in older adults, which often lead to lasting impairments and an increased risk of mortality, are a major public health concern. Hip fracture risk is multi-factorial, affected by the risk of falling, the load acting on the femur, and the load the femur can withstand. This study investigates the influence of impact direction on hip fracture risk and hip protector efficacy. We simulated falls for 4 subjects, in 7 different impact directions (15° and 30° anterior, lateral, and 15°, 30°, 60°, and 90° posterior) at two different impact velocities (2.1 and 3.1 m/s), all with and without hip protector, using previously validated biofidelic finite element models. We found the highest number of fractures and highest fragility ratios in lateral and 15° posterior impacts. The hip protector attenuated femur forces by 23–49 % for slim subjects under impact directions that resulted in fractures (30° anterior to 30° posterior). The hip protector prevented all fractures (6/6) for 2.1 m/s impacts, but only 10% of fractures for 3.1 m/s impacts. Our results provide evidence that, regarding hip fracture risk, posterior-lateral impacts are as dangerous as lateral impacts, and they support the efficacy of soft-shell hip protectors for anterior- and posterior-lateral impacts.

## Introduction

Hip fractures in older adults are associated with long periods of hospitalization, chronic impairment, co-morbidities, depression, decreased mobility, and reduced quality of life.^23,48^ Furthermore, hip fracture patients experience increased mortality rate within the first year after a fracture has occurred.^23,36^ With the elderly population growing, the socio-economic cost associated with hip fractures is expected to grow accordingly.^4,48^

The majority of hip fractures occur as a consequence of falls from standing height or lower.^36,39^ However, only about 1–5 percent of falls result in a hip fracture.^2,22^ As the femur only fractures if the applied load exceeds its load bearing capacity, impact models are an effective way of assessing the biomechanical efficacy of preventive interventions.^2,10,23^ The load bearing capacity of the femur has been shown to decrease with low bone mineral density (BMD),^8,35,48^ be affected by geometrical features of the femoral neck,^8,20,25,34^ and to change with the fall loading configuration.^40^ The load that the femur is subjected to depends on factors specific for the faller, such as soft tissue thickness over the greater trochanter,^3,44^ stiffness,^42^ and shape,^9^ but also on biomechanical aspects of the fall such as impact velocity, impact region, and impact direction.^6,14,28,40,50,51^

While hip protectors have been implemented to attenuate impact forces in case of a fall,^9,29,42,47,49^ clinical studies investigating their effectiveness have revealed conflicting results.^24,45^ One contributing factor may relate to hip protector design. As hip protectors are generally designed for mitigating risk under lateral impacts, they might be less effective under other clinically relevant impact directions, including posterior-lateral falls which have been reported to result in more fractures than lateral impacts.^51^

While biomechanical test standards for hip protectors are emerging,^26^ physical test systems reported in the literature have included varying degrees of biofidelity, and generally simulate the characteristics of a single ‘average’ person. These systems generally allow good control and reproducibility, however, they may be limited in terms of biofidelity (e.g. compliance, inertia, soft tissues properties),^9,29,42^ and the degree to which the impact conditions mimic real-life falls. Recognizing the variance in impact direction, Choi and co-authors rotated the femur-pelvis unit in their test system and found that anterior-lateral impacts resulted in less severe loading of the femur than posterior-lateral impacts.^6^ However, the pendulum impactor used in the study was limited in terms of the rotational degrees of freedom at the hip, only a relatively narrow range of impact directions were examined (− 15° to + 15°), and only unprotected falls were examined (e.g., no impact attenuation interventions). Importantly, the biomechanical effectiveness of hip protectors is also affected by the body shape (e.g. pelvic surface geometry, soft tissue stiffness) of the subjects wearing them.^30^ While mechanical test systems have been adapted to account for subject-specific anthropometrics (e.g. including mass, height, and associated body mass index),^38^ incorporating subject-specific skin surface geometry and soft tissue distribution in the pelvis/thigh regions is challenging to model experimentally and yet likely critical for examining the protective capacity of wearable hip protectors at an individual level.

Recently, we validated subject-specific finite element (FE) models which allow for the simulation of falls to the side.^17^ These models accurately represented ex vivo fall simulations with respect to impact force (RMSE = 10.7%), stiffness (RMSE 12.9%), and fracture outcome (10 out of 11). Furthermore, these FE models allow for the analysis of internal forces^15^ and falls with different impact conditions.^17,19^ Due to the detailed representation of bone and soft tissue geometries and material properties, these models are ideal for virtual exploration of the effect of different impact conditions due to fall and subject-specific characteristics, as well as investigate preventive measures in a paired comparison.

Thus, the aim of the present study was to use these novel computational models^17^ to quantify the influence of fall direction and hip protector padding on impact dynamics in the proximal femur. We hypothesized that: (1) posterior-lateral impacts would lead to as many fractures (from a biomechanical point of view) as lateral impacts; and (2) hip protectors would be less effective in attenuating impact forces and reducing fracture risk in anterior- or posterior-lateral falls compared to lateral impacts. A secondary goal was to compare simulation output from bone models which incorporated linear vs. non-linear stress-strain responses.

## Materials and Methods

### Hip Protector FE Model

**Figure 1:**
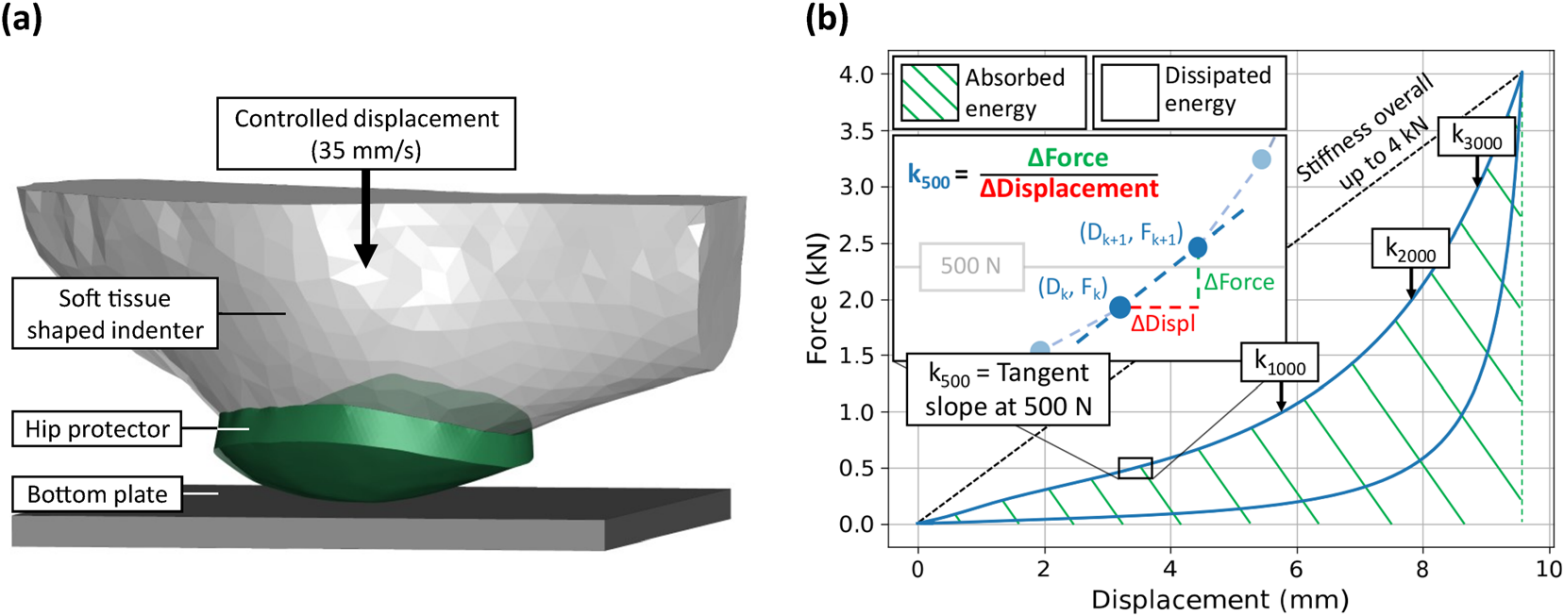
(**a**) Overview of computational hip protector compression test. (**b**) Force-displacement curve resulting from the compression test with extracted force-deflection variables. Depicts the calculation of tangent slope at 500 N (*k*_500_) in detail, as well as where the remaining tangent slopes where measured. Overall stiffness represented through dashed line. Absorbed and dissipated energy represented through colored areas.

Based on the shape and properties of commercially available hip protectors from a previously published study,^29^ we developed a generic hip protector model. The pad dimensions were defined according to the median values for length, width, and thickness of eleven soft-shell hip protectors, resulting in an elliptically shaped hip protector pad with dimensions of 170.0 mm, 150.0 mm, 14.5 mm, respectively. The mechanical characteristics for the pad were calibrated to match the mean mechanical response of the eleven hip protectors. Specifically, an FE model of the hip compression test setup used by Laing *et al*.^29^ was constructed (ANSA 20.1.2, Beta CAE Systems, Switzerland) (Fig. 1a). The hip protector pad was centered over the most lateral point of the greater trochanter and morphed to conform to the indenter shape, which represented the hip-region surface contours of a thin female who would be at elevated fracture risk based on body mass index (BMI = 15.4 kg/m^2^).

The indenter was meshed with 1st-order solid tetrahedral elements (LS-Dyna, Livermore, USA: element formulation (EF) = 13), with a target element edge length of 15 mm. Material properties were linear elastic representing the properties of dental stone (*E* = 2 GPa, $\upnu$ = 0.3, $\uprho$ = 1.00E-6 kg/mm^3^). A 15 mm thick support plate was modelled with three layers of hexahedral elements (EF = 1) with 5 mm edge length and material properties representative of steel (*E* = 210 GPa, $\upnu$ = 0.3, $\uprho$ = 7.85E-6 kg/mm^3^). Based on a mesh convergence analysis, the hip protector pad was discretized with first order tetrahedral elements with an average element edge length of 3 mm (EF = 13), which resulted in an overall stiffness and energy error of less than 2% when compared to a mesh with 1 mm edge length. A material model for highly compressible low-density foams was chosen to represent the mechanical properties of the hip protector (MAT_057, *E*_tension_ = 0.002 GPa, $\uprho$ = 1.00E-7 kg/mm^3^, HU = 0.05, SHAPE = 5). The stress-strain curve and hysteresis input for this material was the result of the reverse engineering process described below.

The bottom surface of the support plate was translationally fixed. The hip protector pad was tied to the indenter surface, while the interaction between the support plate and the hip protector was modelled as frictionless. Reaction forces were measured at the contact between the hip protector pad and the force plate, and indenter displacement recorded. Congruent with Laing *et al*.,^29^ a constant displacement rate of 35 mm/s was used to compress the hip protector to a peak force of 4.0 kN, followed by unloading at the same rate. While the loading rate during the impact phase of an in-vivo sideways fall is dynamic in nature, this approach was sufficient for characterizing hysteretic properties, and importantly, leveraged previously-collected experimental data from 11 commercially-available products to enhance the external validity of the model developed. Hip protector stiffness was measured as the tangent slope at specific points within the force-displacement curve (0.5, 1.0, 2.0, and 3.0 kN) (Fig. 1b). Additionally, the overall stiffness up to 4.0 kN, the stored energy up to 4.0 kN, and the absolute and relative dissipated energy were calculated. We manually calibrated the curve-based material input for the generic hip protector to be within one standard deviation of the experimental results reported for the previously mentioned 11 hip protectors.

### Subject-Specific FE Models for Fall Simulation

The FE model of the hip protector pad was incorporated into pre-existing validated subject-specific FE models for the simulation of sideways falls.^17,18^ In the current study, we used four subject-specific FE models (two females, two males), with different body anthropometrics, bone strength, and experimental fracture outcome^16^ (Table 1).

**Table 1:** Subject summary for the sideways-fall FE models.

Subject	Age (years)	Height (m)	Specimen mass (kg)	BMI (kg/m^2^)	*T*_ST_ (mm)	aBMD_total hip_ (g/cm^2^)	*t* score
H1389 (f)	88	1.63	40.8	15.4	19	0.532	− 3.36
H1395 (f)	67	1.58	99.8	40.2	76	0.711	− 1.82
H1399 (m)	85	1.83	63.5	19.0	10	0.608	− 2.70
H1402 (m)	70	1.75	68.1	22.2	14	0.867	− 0.47
Mean	77.5	1.7	68.1	24.2	29.8	0.7	− 2.1
Std. Dev.	10.5	0.1	24.3	11.0	31.1	0.1	1.2

BMI is the body mass index calculated as the subject mass divided by the subject height squared. T_ST_ is the thickness of soft tissue over the greater trochanter. aBMD_total hip_ is the areal bone mineral density of the proximal femur. t-score is the defined as the number of standard deviations the bone mineral density (BMD) is lower than the BMD of an average healthy 30-year-old adult. A t-score below − 1.0 is considered as low and below − 2.5 is defined as osteoporotic

Details of the subject-specific FE models have been reported by Fleps and colleagues,^15,17^ but are briefly described here for clarity. Pelvis and femur geometry were segmented from calibrated CT scans (scan parameter: 120 kVp, 200 mAs, voxel size: 0.78 mm × 0.78 mm × 0.3 mm, phantom: QC1, Scanco Medical), and material properties were applied based on grey scale values.^11^ The subject-specific soft tissue surrounding the femur and pelvis was modelled as ballistic gelatine using a hyperelastic Frazer-Nash rubber material model. The material properties and density were in between adipose and muscle tissue.^16,18^ Two material models were implemented for bone: (i) with a linear stress-strain response (FEMs_lin_), and (ii) with a non-linear response (FEMs_non-lin_), which enabled the prediction of bone failure.^11,17^ The non-linear material modelling strategy was validated in two ways. Simulations of isolated femur loaded in a drop tower resulted a RMSE of 12.8% for the femur fracture force (regression slope: 0.97). The set of second validation experiments simulated unprotected sideways fall impacts of cadaveric pelvis-femur units that were embedded in surrogate soft tissue and mounted on lower leg surrogates. The finite element models accurately predicted the peak impact force (RMSE = 10.7%), stiffness (RMSE = 12.9%), and fracture outcome (10 out of 11) for impact without injury, with femur fractures, and with pelvic fractures. The linear material model for these FE models did not include any bone failure allowing us to simulate the full load acting on the femur during such an impact, which is independent of the femoral fracture force. The non-linear material model included a description of tissue yield, ultimate strength and softening in tension and compression, as well as a dependency on loading rate. This material implementation allowed us to estimate if the femur would break during the simulated impact and if they break then at which force. While bone material models with linear stress-strain responses have been shown to have reasonable fracture prediction capacity in some applications,^13^ we implemented both as a means to gather insights into differential impact dynamic features and compared the output from each across our impact configuration and hip protector conditions.

**Figure 2:**
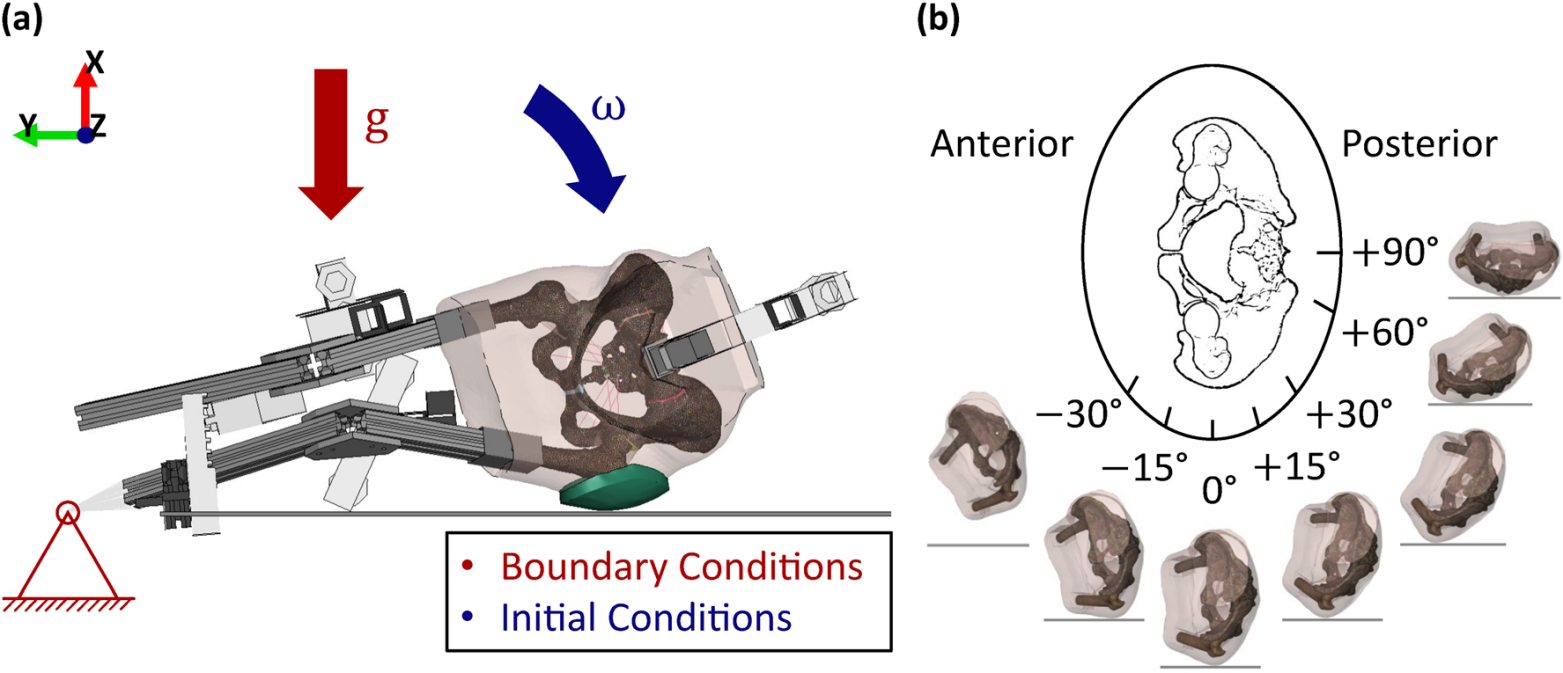
(**a**) Biofidelic FE model, with the generic hip protector pad added (in green). Initial conditions highlighted in blue, boundary conditions highlighted in red. Global coordinate system indicated in top left-hand corner, with the *X*-axis pointing upwards, normal to the impact surface, and the fall motion occurring within the *XY*-plane. The *Z*-axis is pointing outwards, normal to the plane of motion.^15^ (**b**) Visual representation of the different impact directions that were simulated.

The boundary conditions for FE model validation are illustrated in Fig. 2a. Gravity (9.81 m/s^2^) was applied to the entire system in a negative global X-direction. The “foot point” of the model was translationally constraint. The initial position of the system represented a time point just before first contact of the subject with the impact surface. An initial rotational velocity around an axis parallel to the z-axis and directed through the foot point was applied to the whole model. No further constraints were applied to the system.

### Fall simulations

Seven impact directions were modelled for each subject: − 30°, − 15°, 0°, + 15°, + 30°, + 60°, and + 90°, where 0° represents a lateral impact, negative angles represent anterior impact directions, and positive angles represent posterior impact directions (Fig. 2b).

Two transformations were applied to create the initial conditions of the simulations. First, to introduce the different impact angles, the subjects were rotated around an axis going through the foot point and the center of the femoral head. Second, the subjects were rotated within the plane of motion of the original experiments (*XY*-plane) around an axis which intersected with the foot point. A subject position right before impact with a distance (in global *X*-direction) of 0.5–1.5 mm between the impact surface and the soft tissue was chosen, except for 30° anterior impacts, where the initial position of the subjects had to be modelled mid-fall to avoid intersections between the ipsilateral knee and the impact surface. The initial velocity applied in these simulations was adjusted to balance the additional potential energy with a reduction in initial kinetic energy.

We applied two different impact velocities to the FE models. First, an impact velocity of 3.1 m/s (*v*_impact,3.1_), in accordance with the experimental testing representing a severe fall from standing height and second, an impact velocity of 2.1 m/s (*v*_impact,2.1_), which more closely represents an average impact velocity resulting from a fall of an older adult.^7^

FE models for all subjects were run for all impact directions, at both *v*_impact,3.1_ and *v*_impact,2.1_, using the non-linear bone material to evaluate failure outcomes. FE models with linear elastic bone material properties were run for all directions at *v*_impact,3.1_ to evaluate dependencies between impact direction and femoral loading.

Finally, to investigate the effectiveness of the hip protector at preventing hip fracture, all impact conditions were run with and without the generic hip protector. The hip protector was implemented by fixing it onto the soft tissue, just below the most lateral point of the greater trochanter.

### Post processing of data

Peak forces were quantified at the impact surface (“impact force”), at the greater trochanter (“greater trochanter force”) and at the acetabulum (“femur force”). All force-time vectors were filtered using a fifth order low-pass Butterworth filter with a cut-off frequency of 2000 Hz (output frequency = 20,000 Hz). For the 60° and 90° posterior impacts, peak femur and greater trochanter force was identified at the time-point where peak impact force occurred.

Failure of the bone was assessed by quantifying the first and third principal engineering strains in the solid elements that form the surface of the femur analogue to the study of Fung *et al*.^19^ Strains were averaged over the integration points of each element to calculate a single strain tensor for each element. The failure thresholds for cortical and trabecular bone, which correspond to the strains at the ultimate stress in our material model, are shown in Table 2. As a result of the material mapping procedure, elements with material properties corresponding to trabecular bone could be part of the elements that form the femur surface in areas with thin cortices. Any elements of the femur within a 5 mm distance in X-direction from the node closest to the impact surface, as well as elements on the femoral head were excluded in the failure evaluation.

**Table 2:** Failure strain thresholds for each bone type in the femur.^19,21^

Bone type	Strain type	Strain threshold (%)
Trabecular	1st principal strain	1.4
Trabecular	3rd principal strain	− 2.0
Cortical	1st principal strain	2.8
Cortical	3rd principal strain	− 5.9

Based on strains, each FEMs_non-lin_ outcome was classified into one of three categories: “No Femur Fracture”, “Potentially Damaged Femur”, and “Fractured Femur”. A simulation outcome was characterized as No Femur Fracture, when neither compressive nor tensile strain exceeded the strain thresholds. The Potentially Damaged Femur category was created for unclear cases, where compressive or tensile strains exceeded thresholds in less than 5 connected surface elements. Finally, the Fractured Femur category represented cases that based on our assessment would more clearly result in a fracture according to the simulation result, represented by growing cracks on the surface of the FE models and a drop in force response. This was found generally to occur for FE models with strain thresholds being exceeded in more than 5 connected surface elements, effectively excluding isolated highly strained elements.

To evaluate the effectiveness of the hip protector, the force attenuation between the FEMs_lin_ with and without hip protector was calculated for peak forces at the impact surface, at the greater trochanter and at the acetabulum by using the following equation:

1 $$\mathrm{Force\,attenuation}=\frac{{\mathrm{F}}_{\mathrm{peak},\,\mathrm{without\,hip\,protector}}-{\mathrm{F}}_{\mathrm{peak},\,\mathrm{with\,hip\,protector}}}{{\mathrm{F}}_{\mathrm{peak},\,\mathrm{without\,hip\,protector}}}\cdot 100\mathrm{\%}$$

In addition, the peak femur forces were used to calculate a fragility ratio (FR), which is the ratio between the peak forces according to the FEMs_lin_ simulations and its corresponding FEMs_non-lin_ simulation.^12^ The FR is similar to the load-to-strength ratio, which is frequently used to assess structural integrity, with the distinction that the femur force in the non-linear simulation is only the femoral strength in simulations that indicate a fracture. As a result, the lower bound of the FR is 1. The larger the FR, the more severely has the load that would be applied to the femur in a given impact surpassed the force that the femur can stand in this loading configuration.

## Results

**Figure 3:**
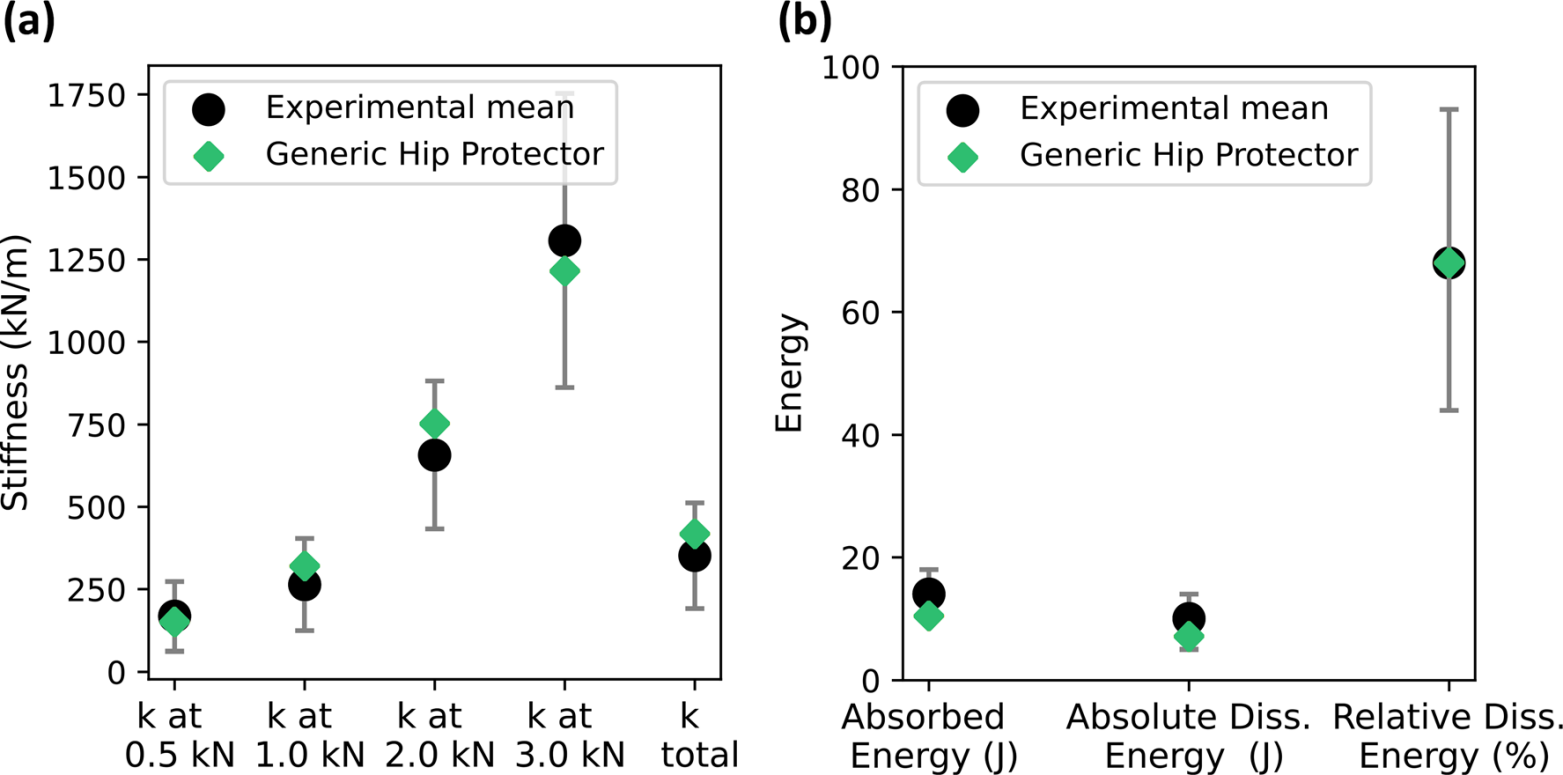
Force-deflection variables of the generic hip protector after material calibration compared to the experimentally tested 11 soft shell hip protectors reported by Laing *et al.*^29^ (**a**) Tangential stiffness values and overall stiffness up to 4.0 kN. (**b**) Absorbed energy and absolute and relative dissipated energy. Results for the generic hip protector are depicted by black diamonds. Mean target values from experimental results are depicted by green dots and $\pm$ 1 standard deviation is represented by grey whiskers.

The material calibration results are depicted in Fig. 3, which shows that all force-deflection properties are within one standard deviation of the mean experimental results reported by Laing *et al*.^29^ (Fig. 3).

**Figure 4:**
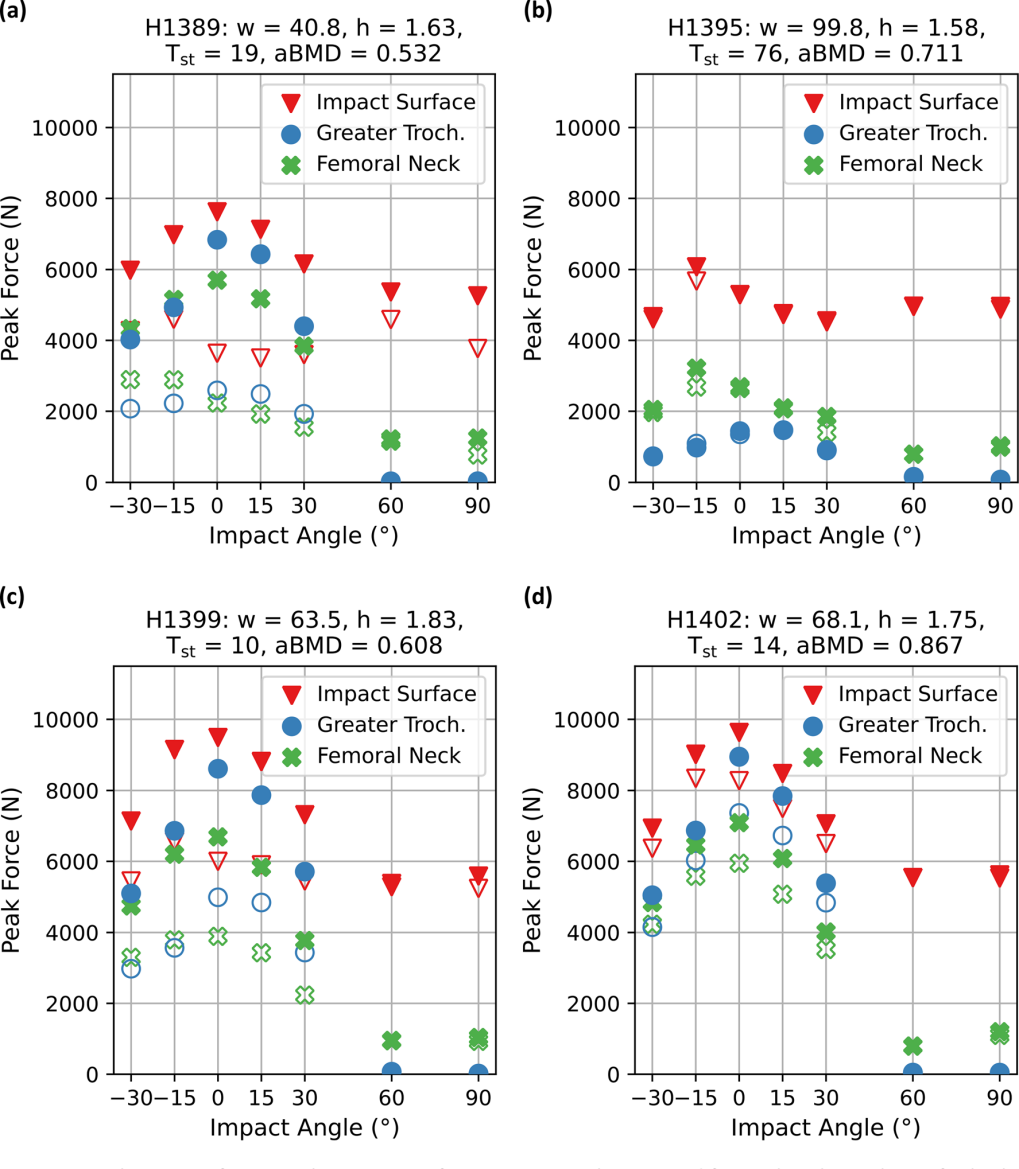
Peak reaction forces at the impact surface, greater trochanter, and femoral neck are shown for both, linear and non-linear material models with an impact velocity of 3.1 m/s and plotted against impact angles for all subjects. Impact forces are depicted with red triangles, greater trochanter forces with blue circles, and femoral neck forces with green crosses. The linear conditions are represented by filled markers, whereas the non-linear conditions are represented through empty markers. Impact angles are defined with respect to the frontal plane, where anterior angles are defined in negative direction and posterior angles in positive direction. Subject characteristics: *w* = weight of specimen in kg, *h* = height of subject in m, *T*_st_ = soft tissue thickness over the greater trochanter in mm, aBMD = areal bone mineral density in g/cm^2^. (**a**) Subject H1389. (**b**) Subject H1395. (**c**) Subject H1399. (**d**) Subject H1402.

For the fall simulations, peak forces were equal or higher for the FEMs_lin_ than for their FEMs_non-lin_ counterpart at all three sites where they were evaluated (Fig. 4). In the FEMs_lin_, the peak impact force was highest in either the lateral or 15° anterior impacts and then decreased with increasing impact angles, in both anterior and posterior direction. The general trend observed regarding the magnitude of the peak force between the three sites was as follows: For the lateral and posterior-lateral impacts (0° to +30°), the peak force was highest at the impact surface, then decreased at the greater trochanter and was lowest in the femoral neck. In the anterior-lateral impacts, the peak forces at the greater trochanter dropped below the peak forces in the femoral neck. The same was true for the 60° and 90° posterior conditions, though in this case, the peak forces at the greater trochanter were almost zero.

In the simulations that were used to analyze femoral loading (FEMs_lin_), we observed similar trends as in the simulations that included bone failure (FEMs_non-lin_), though there were some differences between subjects. H1389, a female subject with low soft tissue thickness (*T*_st_), and lowest aBMD, experienced the most femoral fractures and showed the greatest difference in magnitude of peak forces between its FEMs_non-lin_ and its FEMs_lin_. H1395, a subject for which no femoral fractures were observed and with the largest *T*_st_, showed almost equal peak force response for the two material models. An exception to this was the 15° anterior condition, which was also the condition in which we observed a femoral shaft fracture. Our two male subjects (H1399 and H1402) of similar *T*_st_ but different aBMD, exhibited peak forces of similar magnitude in the FEMs_lin_, but the subject with lower aBMD (H1399), which had fractured the femur experimentally, showed a greater decrease of peak forces in the FEMs_non-lin_ compared to the subject with high aBMD (H1402), which did not fracture the femur.

**Figure 5:**
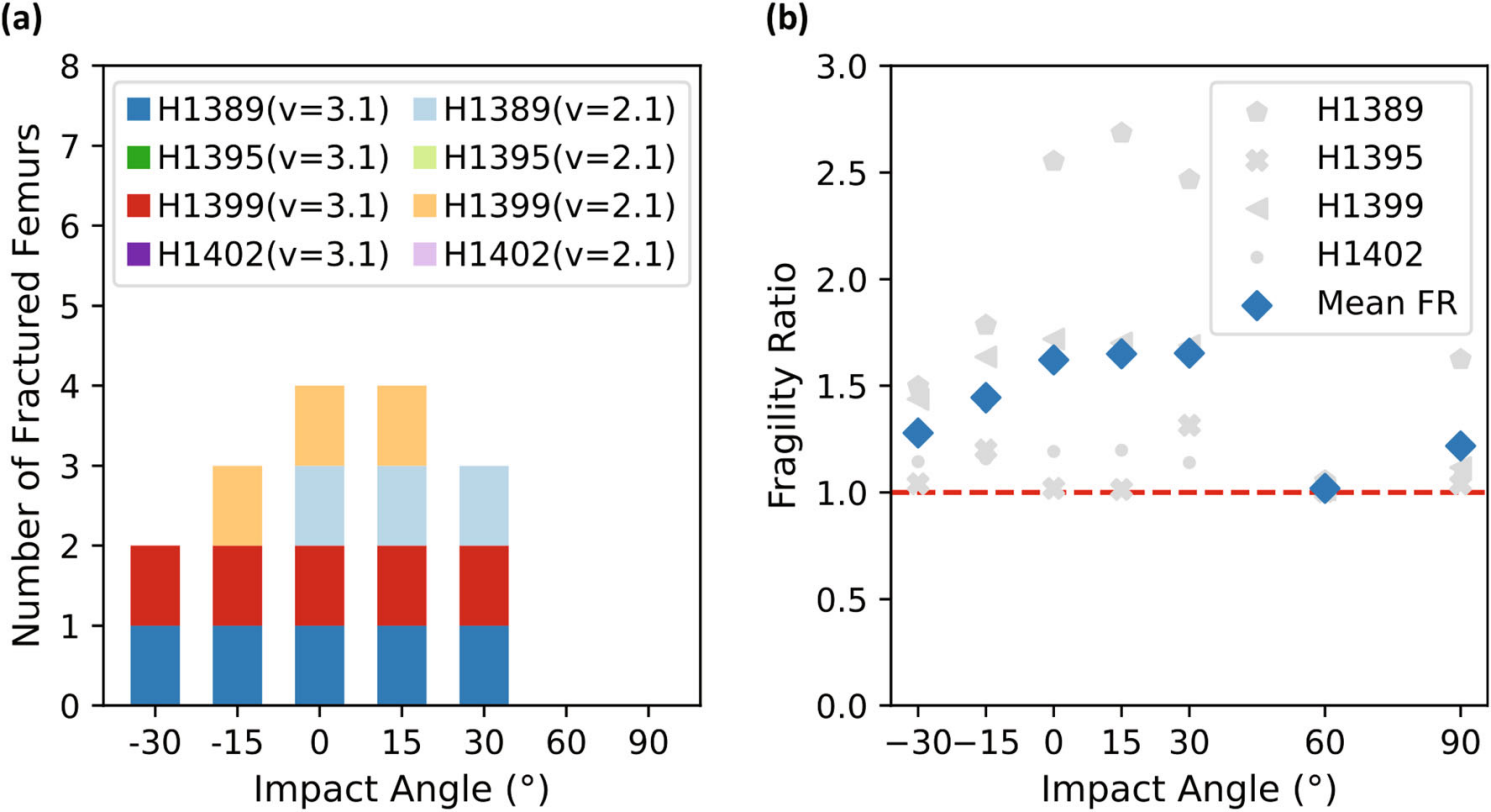
(**a**) Number of fractures vs. impact angles for all simulations without the hip protector. Impact angles are defined with respect to the frontal plane, where anterior angles are defined in negative direction and posterior angles in positive direction. (**b**) Fragility ratio (FR) vs. impact angles for all subjects. Mean FR depicted with blue diamonds.

**Figure 6:**
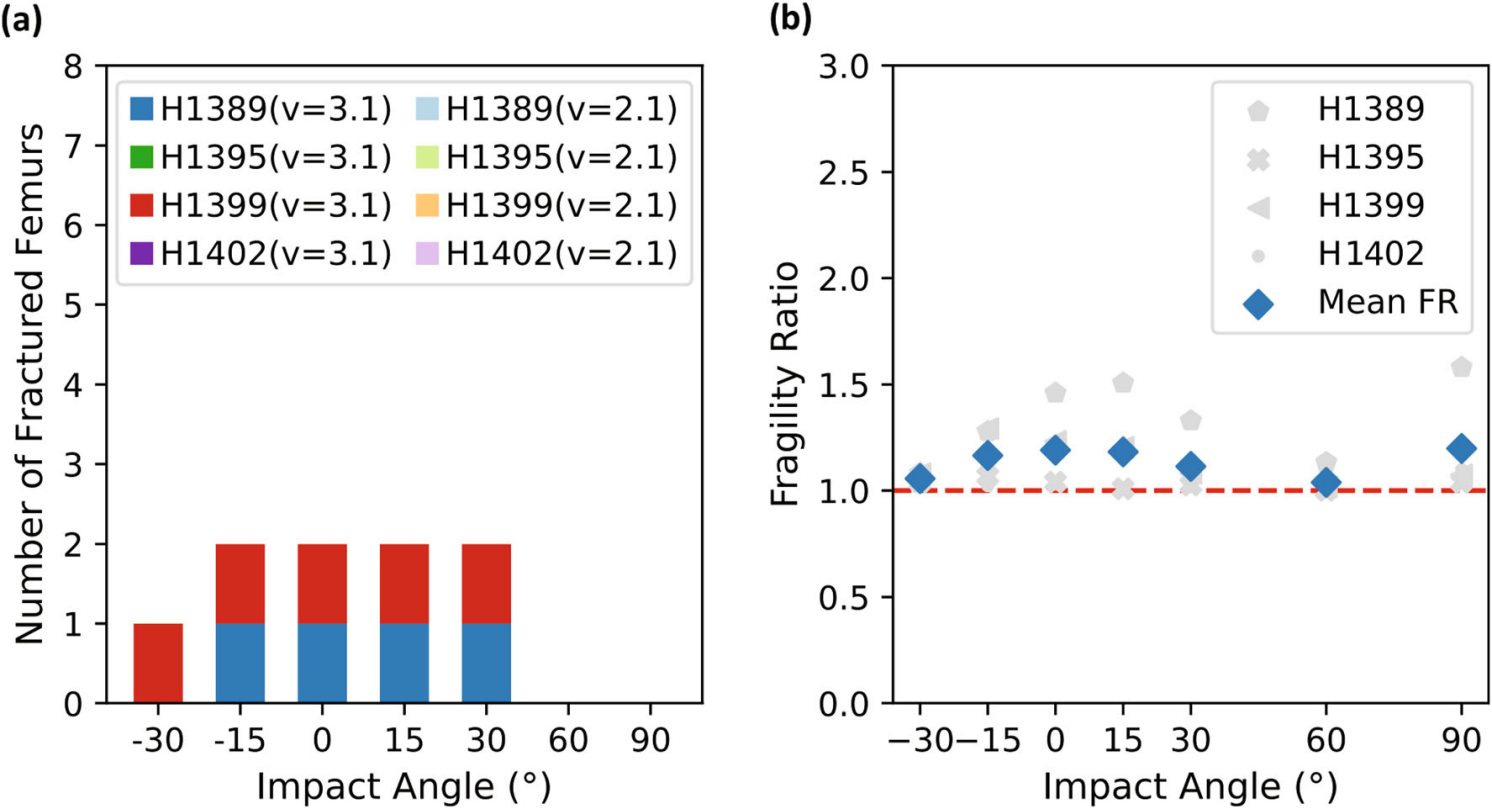
(**a**) Number of fractures vs. impact angles for all simulations with the hip protector. Impact angles are defined with respect to the frontal plane, where anterior angles are defined in negative direction and posterior angles in positive direction. (**b**) Fragility ratio (FR) vs. impact angles for all subjects. Mean FR depicted with blue diamonds.

Out of the 56 simulated impact conditions, we observed 16 femoral fractures from the unprotected falls (Fig. 5a) and 9 in the simulations with the hip protector (Fig. 6a). For both impacts with and without hip protector, most fractures occurred in the lateral (6/25) and in the 15° posterior (6/25) impacts, followed by the 30° posterior (5/25), the 15° anterior (5/25) impacts, and the 30° anterior impacts (3/25). No femoral fractures were observed in the 60° and 90° posterior impacts, although pelvic fractures were seen for subject H1389 (not depicted). We report 10 Potentially Damaged Femurs in the unprotected simulations, quite distributed across subjects and impact angles, and 9 in the protected simulations, of which 8 were attributed to subject H1395. Both, Potentially Damaged and Fractured Femurs were observed for subjects H1389 and H1399, whereas simulations for subjects H1395 and H1402 only showed Potentially Damaged Femur cases and No Fractured Femurs. Subject H1395 showed a shaft fracture at the lateral and 15° anterior impact with *v*_impact,3.1_. The fast impact velocity simulations led to a greater number of fractures (19/25) than those with *v*_impact,2.1_ (6/25). The hip protector prevented 7 femoral fractures, of which 6 were observed in simulations with *v*_impact,2.1_ and one was observed in a simulation at v_impact,3.1_ at a 30° anterior impact.

The mean FR for the unprotected simulations was highest for the 15° and 30° posterior impacts (FR = 1.65 for both but was closely followed by the lateral (FR = 1.62) impacts (Fig. 5b). The inclusion of the hip protector resulted in decreased FRs, especially at the impact angles where most femoral fractures occurred (− 30° to + 30°, Fig. 6b).

**Figure 7:**
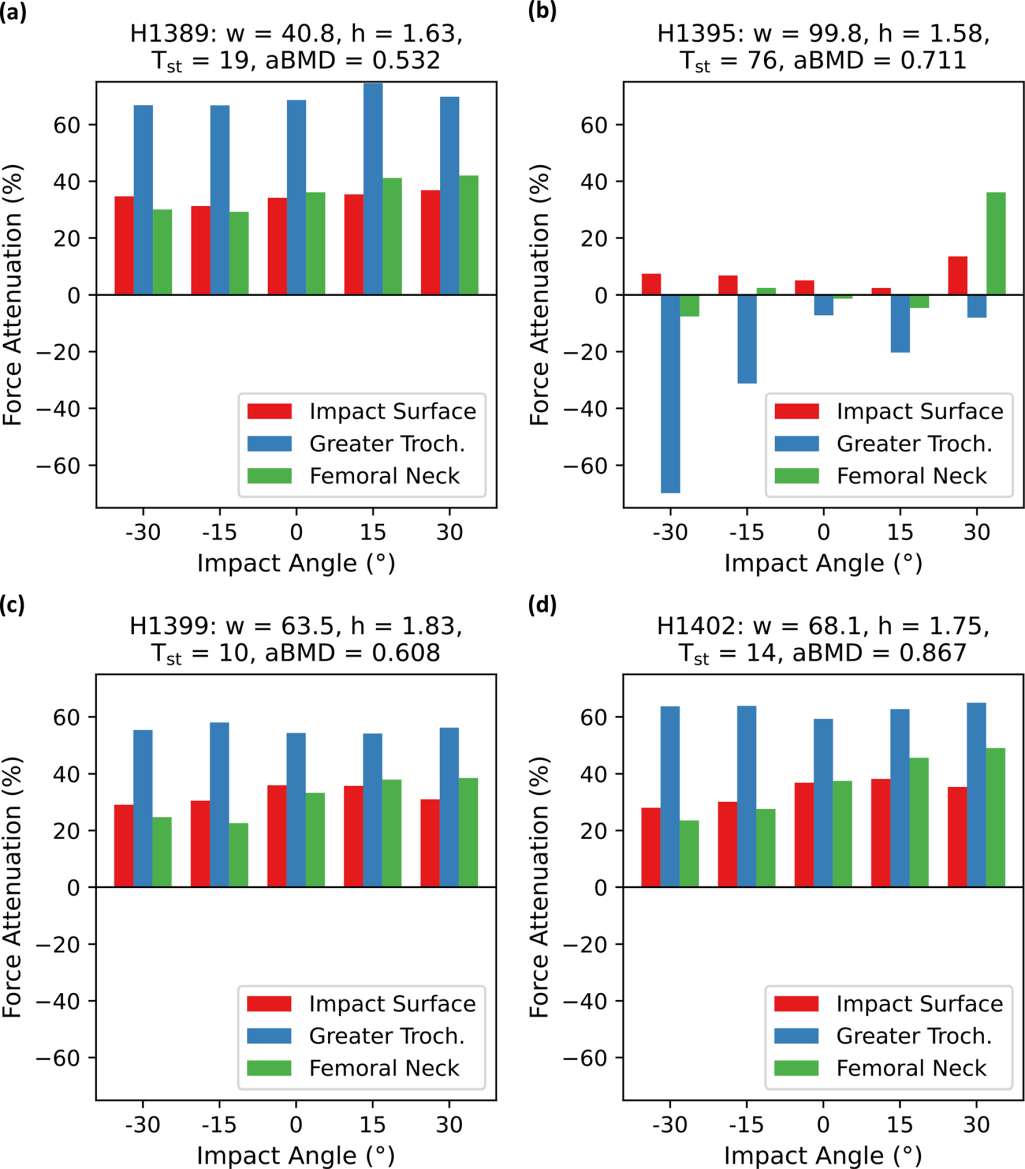
Attenuation of peak force at the impact surface, at the greater trochanter and at the femoral neck through the hip protector in the FEMs_lin_ with an impact velocity of 3.1 m/s. Impact angles are defined with respect to the frontal plane, where anterior angles are defined in negative direction and posterior angles in positive direction. Subject characteristics given: *w* = weight of specimen in kg, *h* = height of subject in m, *T*_st_ = soft tissue thickness over the greater trochanter in mm, aBMD = areal bone mineral density in g/cm^2^. (**a**) Subject H1389. (**b**) Subject H1395. (**c**) Subject H1399. (**d**) Subject H1402.

For the three subjects with low *T*_st_ (H1389, H1399, H1402), the force attenuation through the hip protector was highest at the greater trochanter and varied between 54.1% and 74.5% (Fig. 7). The force attenuation at the impact surface and at the femoral neck varied between 29.1-38.2% and 22.6–49.0%, respectively and tended to increase from anterior to posterior impacts. The subject with the highest *T*_st_ (H1395) showed a different force attenuation pattern (Fig. 7). We observed low force attenuation at the impact surface (2.5–13.6%) and a force attenuation close to zero for the femoral neck, except for the 30° posterior condition, where the attenuation was comparable to the other subjects. At the greater trochanter, the peak forces were higher with the hip protector, ranging between an increase of 7.3 to 69.8%.

## Discussion

The aim of this study was to quantify the influence of fall direction and hip protector padding on impact loading at the hip, and to evaluate hip protector effectiveness under different impact conditions. We found an equal number of fractures in lateral and 15° posterior impacts, suggesting that posterior-lateral falls are at least as dangerous as lateral falls. The lower number of fractures and lower fragility ratios in the anterior-lateral impacts suggest that these are less dangerous for the femur than lateral and posterior-lateral falls. As expected, the force attenuation through the hip protector for anterior-lateral impacts was lower than for lateral impacts. However, contrary to our expectation, the force attenuation through the hip protector was slightly higher for posterior-lateral impacts compared to lateral impact, showing a comparable protective effect for the most dangerous impact directions.

For unprotected impacts, femoral loading and femoral strength both changed with impact orientation. Femoral strength in simulations that resulted in fractures (subjects H1389 and H1399) was highest in 15° anterior impacts and gradually decreased for more posterior impacts. This is consistent with respect to strength magnitude and the influence of loading direction when compared to ex-vivo testing^40^ and computational models^27,41^ of isolated femurs. Patterns in femoral loading were consistent across subjects, with highest femoral loading in anterior-lateral or lateral alignments and decreased loading for more anterior and more posterior impact direction. Femoral strength follows a similar pattern, resulting in similar FR values and number of fractures for lateral, 15° posterior and 30° posterior impacts. FRs were higher for lateral and posterior-lateral impacts compared to anterior-lateral and posterior impacts. This is in agreement with studies reporting real-life fractures captured on video.^51^ No femoral fractures were observed in the 60° and 90° posterior impacts, but pelvic fractures were seen for subject H1389, indicating that these impact directions are likely more dangerous for the pelvis than the femur. This highlights the importance of considering impact specific femoral loading in combination with loading configuration specific femoral strength when evaluating hip fracture risk.

The hip protector decreased the total number of fractures as well as the FR for the dangerous impact angles (− 30° to + 30°) by reducing the impact load and thereby bring it closer to or even below the femoral strength. The force attenuation provided by the hip protector was highest at the greater trochanter, and of similar magnitude at the impact surface and at the acetabular cup for our three low BMI subjects. This indicates that in simulations with the hip protector, the force transmitted through the greater trochanter is reduced and the femur experiences a force transfer through the surrounding soft tissues. Interestingly, in our high BMI subject, we saw an increased peak force at the greater trochanter in the simulations with the hip protector. This is likely a result of the hip protector acting as a force concentrator, since its surface is smaller than the contact area between the impact surface and skin surface for this subject in an unprotected fall. This aligns with previous reports that hip protectors are less effective at attenuating peak pressure for high- compared to low-BMI persons,^5,31^ and can actually increase peak pressure in regions peripheral to the greater trochanter.^31^ This load concentrator mechanism is plausible in the current study as the approximate area of the hip protector simulated (200 cm^2^) is smaller than the contact area that^33^ reports for high BMI individuals during sideways falls (up to 250 cm^2^). This phenomenon is most pronounced in the anterior impacts, where the impact surface of the soft tissue for unprotected impacts is increased because of the thigh coming into contact with the ground. A larger study with an adequate spectrum of body anthropometrics could help distinguish between subjects that might benefit from wearing hip protectors and subjects with a marginal protective effect or even increased fracture risk.

We observed a much higher number of fractures in the *v*_impact,3.1_ vs. *v*_impact,2.1_ simulations. However, little is known about the subject-specific factors that might determine the probability distribution of subject-specific impact velocity. The hip protector pad in our study mostly prevented fractures at *v*_impact,2.1_, and there is evidence that this is a common impact speed in real-life falls.^7^ However, in more severe falls at *v*_impact,3.1_, which is still in the range of observed impact velocities, the force attenuation of this hip protector was insufficient for preventing fractures. This suggest that hip protectors might only provide protection for less severe falls in subjects at high risk of hip fracture. These findings also indicate there may be an opportunity for continued innovation in hip protector design to confer additional benefits at higher impact energies.

Laing *et al*.^29^ reported hip protectors with diverse shape, material, and protective mechanism, whereas our findings are limited to foam-based soft-shell hip protector pads. Moreover, variations in hip protector geometry, material stiffness, and viscoelastic response might influence our findings. In comparison to this variety of hip protectors our generic hip protector model demonstrated an adequate mechanical response that fell within one standard deviation of the measured response of the eleven soft-shelled hip protectors used for the material calibration, providing confidence that the mechanical response of the hip protector that we modelled is conceptually representative of a generic soft-shelled hip protector pad constructed of low-density polyurethane foam. For lateral impacts, the force attenuation provided by our hip protector for the low BMI subjects (33.3–37.5%) was comparable to the force attenuation provided by the 11 soft-shell hip protectors reported in literature (10.1–40.0%) for 3 m/s impacts.^29^ We therefore believe that our hip protector model is a simple and adequate model that can be used to investigate aspects related to the effect of fall characteristics and subject anthropometrics.

The presented impact models for falls in the elderly represent a cost effective, ethical, and versatile tool for the investigation of hip fracture risk under various conditions (impact angle, impact velocity, and the addition of hip protector padding) which are easily implemented. Nevertheless, our fall models have limitations, such as the homogeneity of the soft tissue surrogate, the fact that the upper body is not modelled, the rigid attachment of the masses to the lower limb assembly, and passive musculature, which have previously been discussed in detail.^17^ Moreover, the model validation was done against purely lateral impacts (impact direction 0°, *v*_impact,3.1_). The accuracy when modelling other impact directions and velocities is unknown. However, in contrast to the single degree of freedom impact models that are commonly used in literature,^1,32,43,46^ our modelling approach represents the whole geometry of the soft and hard tissue of the pelvic region, allowing for a realistic stiffness distribution that does not require calibration based on the impact conditions and should therefore not be limited to lateral falls. The models also showed good agreement with low height impact experiments performed with young volunteers, which supports their validity across impact velocities.^17^

The fall posture of the subjects was not changed with respect to the impact angles. Although we consider the current posture as realistic for all simulated impact angles, it is likely that fall postures will vary between fall direction and impact angles in real life, with certain trends being common for certain fall and impact directions.^51^

Our study focused on medium to low BMI subjects (three out of four subjects), because of the increased hip fracture risk for this population group.^3,12,37^ The subject with higher BMI was included to get a sense of the effect of a thicker soft tissue covering the greater trochanter. With the development of automated pipelines to create similar FE models,^12^ future studies could investigate the effectiveness of hip protectors for a diverse spectrum of fall characteristics across a large number of subjects. Such an approach would improve our knowledge of the spectrum of falls for which hip protector designs are protective and it would help us identify individuals that would benefit most from wearing hip protectors. While the current study focused on a single hip protector with properties characterized from experimental trials at one displacement rate, other hip protector designs (considering geometry, materials, rate-dependencies) could be developed and evaluated, allowing for a cost- effective and reproducible assessment under multiple impact scenarios. Furthermore, the effect of hip protector positioning and the conditions at the interface between the hip protector and skin on its effectiveness could be analyzed.

In summary, we found that peak femur loads are highest in lateral falls. However, posterior-lateral falls are at least as dangerous for the proximal femur, due to decreased femoral strength. Posterior impacts did not pose a high risk to the femur. We found no evidence of the hip protector being less effective in posterior- or anterior-lateral falls than lateral ones. However, the addition of the hip protector we modeled was substantially more effective at preventing fractures at the lower impact velocity. Interestingly, for the high BMI subject that was at low risk of hip fracture, we found evidence to suggest that wearing a hip protector could slightly elevate hip fracture risk by acting as a force concentrator. Thus, our results highlight the need for applying hip fracture prevention methods which consider subject- and fall-specific characteristics.
